# Human Papillomavirus Infection during Pregnancy and Childhood: A Comprehensive Review

**DOI:** 10.3390/microorganisms10101932

**Published:** 2022-09-28

**Authors:** Ali Ardekani, Erfan Taherifard, Abolfazl Mollalo, Emadeddin Hemadi, Amirhossein Roshanshad, Reza Fereidooni, Safoura Rouholamin, Mahroo Rezaeinejad, Maryam Farid-Mojtahedi, Maryam Razavi, Ali Rostami

**Affiliations:** 1 School of Medicine, Shiraz University of Medical Sciences, Shiraz 73, Iran; 2 Health Policy Research Center, Institute of Health, Shiraz University of Medical Sciences, Shiraz 73, Iran; 3 Department of Public Health and Prevention Science, School of Health Sciences, Baldwin Wallace University, Berea, OH 44017, USA; 4 School of Medicine, Ahvaz Jondishapur University of Medical Sciences, Ahvaz 63, Iran; 5 Department of Obstetrics and Gynecology, School of Medicine, Isfahan University of Medical Sciences, Isfahan 83, Iran; 6 Department of Obstetrics and Gynecology, Imam Khomeini Hospital Complex, Tehran University of Medical Sciences, Tehran 13, Iran; 7 Department of Obstetrics and Gynecology, Arash Women’s Hospital, Tehran University of Medical Sciences, Tehran 13, Iran; 8 Department of Obstetrics and Gynecology, School of Medicine, Zahedan University of Medical Sciences, Zahedan 98, Iran; 9 Infectious Diseases and Tropical Medicine Research Center, Health Research Institute, Babol University of Medical Sciences, Babol 47, Iran

**Keywords:** HPV, gestation, newborn infants, children, pregnancy complications, neoplasms, retinoblastoma

## Abstract

Human papillomavirus (HPV), the most prevalent sexually transmitted disease worldwide, is the causative agent for several genital and oropharyngeal cancers and a suspected agent for many malignancies. HPV is associated with several adverse health outcomes during pregnancy. Infants are also at risk of HPV infection via different transmission routes: vertically from an infected mother and horizontally through sexual or non-sexual contact with infected individuals. Several HPV manifestations have been identified during childhood, ranging from common skin infections to severe complications such as juvenile recurrent respiratory papillomatosis. This review aims to provide a comprehensive overview of the epidemiology, manifestations, and treatment strategies of HPV infection during pregnancy and childhood. Moreover, we underline the role of vaccination in preventing complications.

## 1. Introduction

As the most frequent sexually transmitted disease (STD) worldwide, human papillomavirus (HPV) has posed a substantial burden to healthcare [1]. In addition to the established causation between HPV and some genital and oropharyngeal cancers, HPV is a suspected agent for lung and esophageal cancers [2]. While over 120 types of HPV have been isolated [3], according to their oncogenic activity, HPV types are divided into high- (16, 18, 31, 33, 35, 39, 45, 51, 52, 56, 58, 59, 68, 73, and 82) and low-risk (6, 11, 40, 42, 43, 44, 53, 54, 61, 72, and 81) types [2,3]. The low-risk HPV types usually cause benign lesions such as warts [4].

The prevalence of HPV among pregnant women in different sampling sites, including the cervix, serum, placenta, urine, or amniotic fluid, was determined in a recent meta-analysis [5]. The study indicated that HPV is more prevalent among pregnant women in less developed countries and pregnant women with certain disorders [5]. Unfavorable pregnancy outcomes such as the preterm premature rupture of membranes and preterm births were also significantly associated with HPV infection [6]. Several studies have investigated the transmission rate of HPV based on the delivery mode [7,8,9] and HPV-related disorders during infancy and childhood [10,11]. In the present review study, we aimed to provide a comprehensive review of HPV infection and its consequences in pregnancy, the possible relationship between HPV and adverse pregnancy outcomes, routes of maternal–fetal HPV transmission, and possible and definite manifestations of HPV in childhood.

## 2. HPV Transmission Routes

While HPV infection is generally considered an STD, the presence of this virus in individuals who never had sexual contact, neonates, or pediatrics implies the presence of other transmission routes [12]. HPV infection is transmitted from mother to infant (i.e., vertical transmission), which could occur either peri-conceptually, prenatally, or perinatally [6,13]. Moreover, HPV infection in infants may be caused via horizontal transmission from peers, parents, and relatives [12]. Therefore, infants are at risk of infection from three main routes [12]: vertical, non-sexual horizontal, and sexual abuse transmissions. 

### 2.1. Vertical Transmission

#### 2.1.1. Peri-Conceptual Transmission 

Vertical transmission of HPV could happen from either parent to their descendants [14]. Vertical transmission from the father may occur around fertilization, either pre-implantation or during implantation, typically known as peri-conceptual transmission [15]. Previous studies have detected HPV in semen that could infect spermatozoon [16]. In a systematic review assessing the prevalence of HPV infections among men, HPV DNA was found in 11.4% of semen samples in the general population and in 20.4% of men referred to infertility clinics [16]. Mechanistic studies have implied that HPV DNA is placed in the head of a spermatozoon [17,18]. Moreover, an infected spermatozoon could serve as a vector to transfer the virus and its genes (i.e., E6/E7 genes) into the oocyte, where these genes are further expressed [17,18]. However, this situation is associated with a high risk for a spontaneous abortion [19]. The vas deferens of healthy men who underwent a vasectomy have also contained HPV DNA [20].

Mothers can also be a source of peri-conceptual transmission [21]. To date, no studies have been conducted to detect HPV DNA in the oocytes; however, studies have reported the presence of HPV in the upper genital tracts, including the endometrium, mucosa of the fallopian tubes, ovarian epithelium, and germinal epithelial inclusions in patients with invasive cervical cancers who had undergone a hysterectomy and salpingo-oophorectomy [22,23]. In a systematic review and meta-analysis of a total of 2280 patients with ovarian cancer, the pooled prevalence of HPV detection was estimated at 15.9% (95% confidence interval (CI): 11–22%) [24]. As the significance of the results is debatable, further studies are required, mainly to discover the presence of the HPV genome within oocytes.

#### 2.1.2. Intrauterine Transmission 

Intrauterine transmission of HPV, also known as prenatal transmission, is among the proposed HPV transmission routes in a fetus [25]. HPV DNA has been detected in placental samples [5,26,27], amniotic fluid [5,9,28], and fetal membranes [29]. In these cases, the fetus could be infected through micro-abrasions in the membranes or through the placenta, from its tissue or the cord blood. In a cohort study on pregnant women, cervicovaginal samples and maternal and fetal placental tissues were obtained. The results showed that about 14 percent of samples tested positive for HPV, and viral particles were found on both sides of the placenta [27]. Furthermore, in most cases, the genotype was concordant with the isolated genotype from cervicovaginal specimens taken from pregnant mothers [27]. In a study by Rombaldi et al. [26], HPV was detected in 24.5% (12 out of 49) of placental samples of women with positive cervicovaginal HPV tests. Concordance between samples from placental tissues and the genital areas of the mothers was observed in 11 pairs. While HPV tests were positive in seven genital/placental/newborn samples, suggesting an intrauterine transmission of HPV. Inoculation of the placenta with HPV could occur as a complication of an ascending infection from the cervicovaginal areas [30]. Discordance in genotypes of HPV DNA could result from the presence of various genotypes of HPV, inadequate sampling, or improper sampling techniques [27]. 

Hematogenous spread from the mother to the fetus via cord blood has also been postulated as a possible HPV transmission route; however, the transmission possibility is relatively low [31]. Viremia has been reported to have no effect on the viral replication cycle of HPV [32]. There is limited evidence regarding HPV transmission through blood. The blood from infected animals has been injected intravenously into two naïve animal models: mouse and rabbit [33]. In this experiment, papillomavirus infections were observed in the tissues of these naïve animals [33]. HPV DNA has been identified in the lymphocytes of peripheral blood samples [34]; however, the potential for activation, replication, or infection warrants further investigations. 

#### 2.1.3. Perinatal Transmission 

During the delivery, direct contact of the fetus with the HPV-infected cells of the genital tract of the mother, mainly the vagina and the cervix, could result in perinatal transmission. The presence of genital warts and positive HPV tests from cervicovaginal samples of the mothers coupled with positive tests from conjunctival, buccal, pharyngeal, and genital swabs of the neonates suggest perinatal HPV transmission [8]. Concordance between the HPV types of maternal and fetal samples lends credence to the vertical transmission theory [35].

In a systematic review and meta-analysis by Chatzistamatiou et al., eight studies containing information on 446 mother–infant pairs were analyzed [8]. This study assessed the type-concordant transmission of HPV, and the pooled perinatal transmission rate was estimated at 25% (95% CI: 20 to 29%). Furthermore, there was a significantly lower relative risk of HPV transmission in participants who had cesarean section than those who delivered vaginally. However, cesarean delivery did not outweigh the risk of neonatal laryngeal papillomatosis [36]. The persistence of infection in an afflicted infant through perinatal transmission has been controversial, as it may be due to contamination or involvement of the top layers of skin, which normally could be shed during the first days of life [37]. In a longitudinal study by Castellsagué et al., neonates with positive HPV DNA tests at birth were followed up with, and the test was repeatedly performed on multiple visits [38]. In more than 80% of neonates, the subsequent HPV tests performed on their 6th week were negative, implying that the cesarean delivery route would not provide complete prevention, negating the routine use of cesarean section in pregnant women with positive HPV tests [39]. A cesarean section may benefit a selected group of mothers with bulky or friable HPV lesions in their genital tract [36]. Table 1 presents the studies that addressed the possibility of vertical transmissions.

### 2.2. Non-Sexual Horizontal Transmission

A body of literature also shows non-sexual skin-to-skin or skin-to-mucosa HPV transmissions [60]. Non-sexual transmission can occur by heteroinoculation, autoinoculation, inanimate objects, or fomites [61]. 

Heteroinoculation is mainly seen among family members, and transmission can occur by kissing and other non-sexual contacts such as changing diapers, bathing, or fondling [61]. Viral persistence in the oral cavity is quite long [62,63]; therefore, oral and (to a lesser degree) hand warts are hypothesized to impact the transmission. In addition to the cutaneous and low-risk HPV types, high-risk HPV types such as types 16 and 18 can be inoculated in this transmission mode [64]. Condyloma acuminatum can occasionally be found on the nipple and areola [65,66]. This may give rise to horizontal HPV transmission from mothers to infants during breastfeeding and can develop between sexual partners without penetrative sex. 

Autoinoculation is the transmission of a virus from one body site to another [13]. For example, scratching the genitalia with an HPV-infected finger or chewing the viral lesion can result in genital and oral HPV, respectively [13,67].

Fomites can also be assumed as a possible way for horizontal HPV transmission [60]. HPV is a stable and heat-resistant virus that remains on the surfaces for a long time and can survive outside living organisms [68]. To date, there has been no definite evidence of HPV infection following the use of an object, but the possibility cannot be ignored. Gynecological equipment has high contamination risks when used to examine a woman with viral shedding [69]. Besides, there is a chance of micro-trauma or micro-abrasions of the genital areas during the examination with the equipment, making the mucosa vulnerable to HPV entry. In a study by Gallay et al., the presence of HPV on non-disposable gynecological equipment such as boxes of gloves, lubricant tubes, colposcope handles, and lamp handles was examined. They found that 18% of the samples were contaminated with HPV [69]. HPV can also be found on surfaces such as common shower rooms, bathroom surfaces, or underwear [67,70,71,72]. De Martino et al. found that foreskins of 6 out of 50 boys without a history of sexual relations tested positive for high-risk types of HPV and suggested shared towels or other objects as possible sources of transmission [73]. However, positive tests for HPV in those objects do not necessarily indicate infection but a detectable level of viral particles. 

### 2.3. Sexual Abuse 

Pediatric sexual abuse is a neglected health concern, especially when the child has mental or physical disabilities to express the issue properly [74,75]. The presence of abnormal or changed behavior and inconsistent or illogical statements by parents or caregivers raise suspicions of child abuse for the physician; however, in many cases, there are no physical findings or associated injuries [76,77]. In these cases, HPV might be transmitted by oral-to-genital, oral-to-anal, genital-to-anal, or genital-to-genital contacts [77]. Unger et al. conducted a multicenter study on 537 children without having a history of consensual sexual contact referred for an evaluation of child sexual abuse [76]. The samples were taken from the urine and external genitalia of both genders for HPV tests. Among all individuals with any evidence of sexual abuse (possible, probable, or definite), the rate of positive HPV tests was 13.7%, while the rate was higher among those with a higher certainty of abuse (possible: 8.4%, probable: 15.6%, and definite: 14.5%); however, genital warts were only observed in 14 children [76]. Other studies have reported an almost similar prevalence of anogenital warts among children who were sexually abused: 1.8% and 1.3% in studies by Ingram et al. and Muram et al., respectively [78,79]. 

A positive test for an STD, such as HPV infection in an infant, should raise suspicion towards sexual abuse, especially if vertical transmission can be ruled out [80]. However, even in children with no evidence of prenatal HPV transmission, interpreting HPV detection in the anogenital areas as sexual abuse is still subject to debate. This might be due to the lack of substantial evidence on the epidemiology of genital warts and positive HPV tests in infants [80]. Infants need high care from their parents or caregivers, particularly in the diaper sites and anogenital areas, resulting in frequent skin-to-skin contact between infants with their caregivers. Besides, moisture and lacerations or fissures can increase the risk of heteroinoculation, especially with cutaneous types of HPV [81,82]. Therefore, detailed history taking, with a particular focus on perinatal history and performing a meticulous physical examination on the infants and the caregivers, could be beneficial for determining the transmission route. 

## 3. Pregnancy-Related Complications

The prevalence of HPV among pregnant women is higher than in non-pregnant counterparts [5]. According to a recent analysis, the global prevalence ratio of HPV infection in pregnant women with human immunodeficiency virus (HIV) and pregnant women with pregnancy-related disorders were respectively 3.31 and 2.35 compared to healthy pregnant women [5]. Given that pregnancy is a state of mild immunosuppression [83], the manifestations of HPV may be more severe in pregnant women [84]. Genital warts may become larger and require treatment after the first trimester [85]. For small warts, treatment is usually not required. In the case of annoying symptoms or large lesions, cryotherapy should be performed as the first-line treatment, and laser remains the second-line therapy [85]. Routine treatment options such as 5-fluorouracil, podophyllin, and interferon should not be used during pregnancy [86]. The Centers for Disease Control and Prevention (CDC) does not recommend HPV vaccination during pregnancy [87]. A prelicensure trial among 172 women vaccinated within 30 days of the estimated conception date found a higher rate of spontaneous abortion after 9-valent HPV vaccine exposures (20%) compared with 4-valent HPV vaccine exposures (9.2%) [88]. However, numerous studies have indicated no association between HPV vaccination and adverse pregnancy outcomes [89,90,91,92,93]. Still, the current guidelines are against HPV vaccination during pregnancy, and further investigations are ongoing [87].

**Figure 1:**
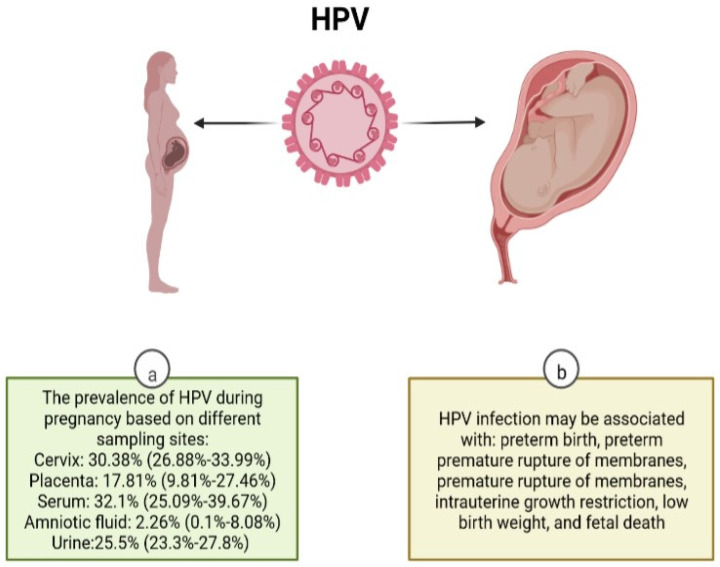
Manifestations of HPV in pregnancy. (**a**) Information adapted from Ardekani et al. [5]. (**b**) Information adapted from Niyibizy et al. [6]. Created with BioRender.com, accessed on 6 July 2022.

Another conflicting entity about HPV infection during pregnancy is the possible relationship of HPV with adverse pregnancy outcomes [6,94,95]. The global prevalence of HPV in placental samples is estimated to be as high as 32.1% (95% CI: 25.09–39.67%), whereas the prevalence of HPV in amniotic fluid samples is estimated to be as low as 2.26% (95% CI: 0.1–8.08%) [5]. A recent comprehensive meta-analysis [6] based on 36 studies has concluded that HPV infection during pregnancy is associated with preterm birth (adjusted for age odds ratio [aOR]: 1.50; 95% CI: 1.19–1.88), preterm premature rupture of membranes (aOR: 1.96; 95% CI: 1.11–3.45), and premature rupture of membranes (aOR: 1.42; 95% CI: 1.08–1.86). Moreover, fetal death (aOR: 2.23; 95% CI: 1.14–4.37), low birth weight (aOR: 1.91; 95% CI: 1.33–2.76), and intrauterine growth restriction (aOR: 1.17; 95% CI, 1.01–1.37) were significantly associated with HPV infection during pregnancy. However, given the suboptimal bias control, the results should be interpreted cautiously. The mechanisms through which HPV infection influences pregnancy outcomes have been suggested to be through alterations in cervical competence, changing the vaginal microbiota, placental infection, amniotic fluid infection, secondary to cervical manipulations (for the treatment of HPV during the pregnancy) and semen infection [5,6,95,96,97,98]. Figure 1 summarizes the prevalence of HPV in different specimens of pregnant women and related complications with HPV infection in pregnant women. 

## 4. HPV in Childhood

**Figure 2:**
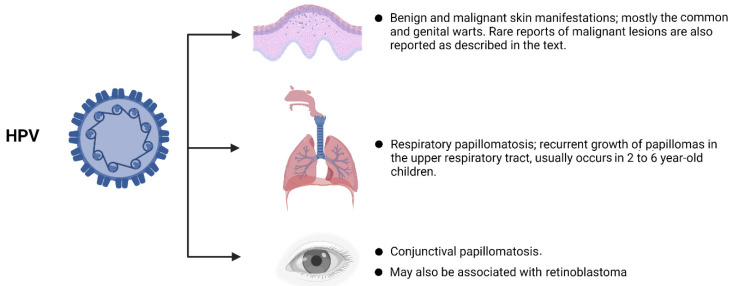
Manifestations of HPV in childhood. Created with BioRender.com, accessed on 6 July 2022.

As screening for HPV among children is not a routine practice, the HPV data in infancy and childhood are inadequate for epidemiological inferences. A well-designed longitudinal study from Finland indicated that the percentage of offspring born to HPV-seronegative mothers and seroconverted to HPV-positive results was 21%, 38%, and 21% at 12, 24, and 36 months of follow-up. This indicates that they acquired HPV infection during their lifetime [99]. For HPV-seropositive mothers, seropositivity in the offspring ranged from 9–25%, 8–38%, and 0–33% based on different HPV serotypes at 12, 24, and 36 months of follow-up, respectively [99]. The seroprevalence of HPV among children of various ages was 15–44% in a review study [100]. In addition, the presence of HPV in the foreskins of asymptomatic children was 17.3% (95% CI: 0.8 to 46.3%) [101]. To our knowledge, there is no global estimate of HPV prevalence in children; however, the current reports indicate a relatively high prevalence of HPV in infancy and childhood, calling for immediate preventive measures. A wide range of definite and possible manifestations in infancy and childhood have been reported for HPV infection (Figure 2) as following: 

### 4.1. Skin Lesions

One of the most common presentations of HPV is verruca vulgaris, also known as the common wart [102]. Wart is usually a solitary rough-surfaced papule that commonly occurs on the back of hands, fingers, or knees but can occur on any part of the body, including the oral cavity and genitalia [103,104]. The common wart is predominant in school-age children [105]. 

Condyloma acuminata are the warts of the anogenital region [61] that usually present as hyper-pigmented plaques or papules, occasionally with a cauliflower-shaped appearance. They are usually in the anogenital region but may less commonly occur in the oral cavity [106]. Due to the long incubation period of HPV (three weeks to eight months) [107], determining the transmission route is difficult. However, the older the child becomes, the less likely vertical transmission is. Thus, screening for sexual abuse should be considered in all cases of anogenital and oral warts, first presenting in children older than three to four years [106]. In addition, differential diagnoses should be considered in case of similar presentations, such as molluscum contagiosum, benign nevi, infantile perianal pyramidal protrusion, and syphilis condylomata [108,109,110,111]. A study reported that six out of eight children who did not receive treatments experienced spontaneous resolution of condylomas [112]. Given the self-resolution of the lesion in most cases, a “Watch-and-Wait” treatment strategy has been suggested, especially for lesions that appeared for less than two years. Several interventional (e.g., laser, cryotherapy, cauterization, and surgical removal) and non-interventional (e.g., Podophyllotoxin, Imiquimod) options are available. However, children are normally less cooperative with interventional therapies, so the non-interventional choices are the first-line treatment [113]. 

Skin cancers are rare in children, and even rarer are HPV-related carcinomas of the skin in children [114]. Bowen’s disease, a type of squamous cell carcinoma (SCC) in situ, has been associated with HPV in immunodeficient children [114].

### 4.2. Mucosal Lesions

Oral squamous papilloma is the most common benign oral lesion associated with HPV in children and adults [115]. They are exophytic projections which may appear red, pink, or white depending on the degree of keratinization [116]. HPV DNA was detected in up to 68% of oral squamous papillomas [117]. Heck’s disease, also known as Focal Epithelial Hyperplasia (FEH), is an uncommon, benign oral disorder associated with infection with HPV types 13, 32, or both [103,118]. It is clinically manifested by multiple small papules and white-to-pinkish (mucosa-colored) plaques in the oral cavity, with a preference for the lower lip. It is most common in children and young adults and has a racial predominance in Eskimo and Native American populations [103,118].

Malignant mucosal lesions are uncommon in children, as the occurrence may take about 5 years (in immunodeficient individuals) up to 30 years following HPV infection [119]. While there is no report of HPV-related neoplasms of the cervix of children, cervical dysplasia, preneoplastic, and perhaps even neoplastic lesions can occur more commonly in immunodeficient children or adolescents [120]. In general, oropharyngeal cancers in children are sporadic; however, several cases of HPV-associated SCC in the oral cavity and oropharynx of children have been reported [121,122,123]. 

### 4.3. Juvenile Recurrent Respiratory Papillomatosis 

Juvenile onset recurrent respiratory papillomatosis (JRRP) is a rare disease mainly caused by HPV 6 and 11 [124]. The disease presents as the recurrent growth of papillomas in the upper respiratory tract, usually in children 2 to 6 years old [125]. Patients with JRRP may exhibit hoarseness (most common presentation), a weak cry, choking episodes, and a failure to thrive [126]. Visible external genital warts in the mother have been strongly associated with JRRP [127,128]. A majority of children with JRRP were those who delivered to young mothers through the vaginal canal, firstborn children, and those who delivered to unvaccinated mothers [129]. Not all infected with HPV 6 and 11 develop JRRP, and the prevalence of JRRP is much lower than the reported prevalence for HPV 6 and 11 [5,130]. This raises the hypothesis of immunosuppression or genetic susceptibility in patients with respiratory papillomatosis. Certain human leukocyte antigens (HLA), namely, HLA- DQB1*0201, DQB1*0202, and DRB1*0301, are associated with severe recurrent respiratory papillomatosis, proposing a regulatory role for these alleles [131]. In addition, killer cell immunoglobulin-like receptors (KIR) regulate the natural killer (NK) cell response against viral infection. Individuals with more severe recurrent respiratory papillomatosis typically lack activating KIR genes 3DS1 and 2DS1 [132]. 

Owing to the changing nature of the disease, its management depends on a case-by-case basis. Some children exhibit modest symptoms with spontaneous remission, while others need several interventions due to rapid disease development [125]. A possibility of lung involvement exists in 3.3% of recurrent respiratory papillomatosis cases [133], which can adversely affect the patients’ quality of life [134]. The repeated surgical removal of papillomas is a widely accepted approach for treatment in symptomatic cases [135] to debulk the papilloma while preserving intact laryngeal tissue. In addition, a number of adjuvant therapies have been proposed to increase the symptom-free intervals [136], such as indole-3 carbinol [137], HPV vaccination [138], bevacizumab [139,140], interferon-α [141], cidofovir [142], programmed cell death protein 1 (PD-1) inhibitors [143], celecoxib along with erlotinib [144,145], and antivirals [146,147].

### 4.4. Retinoblastoma

Retinoblastoma (RB) is the most prevalent intraocular malignancy in children [148]. Previous efforts have identified possible etiologies for sporadic RB [149]. It is hypothesized that due to the higher prevalence of sporadic RB in less developed areas with poorer socioeconomic status, infections may be a possible culprit for the sporadic form of the malignancy [150]. HPV has been notorious for its oncogenic properties and is linked to several malignancies [151]. The E7 protein produced by the high-risk HPV types 16 and 18 bind more avidly to the RB protein than those encoded by low-risk types [152]. The binding, interaction, and subsequent inhibition of the RB protein by E7 is the suggested theory for the carcinogenesis of high-risk HPV types [153]. As RB tumor suppressor gene inactivation has been linked to a range of malignancies, including RB, the hypothesis of the possible role of HPV in RB development has strengthened. Therefore, several studies have evaluated the prevalence of HPV in the intraocular samples of patients with RB [150,153,154,155]. HPV has been detected in RB samples from 0% to 82% of the study participants in different investigations [150,153]. This wide range of positivity for HPV has raised suspicions about its effect on RB development. While some studies support the relationship between HPV and RB progression [155,156,157,158], other studies, especially those in which the HPV prevalence in cases was less than the normal population or controls, refute the hypothesis of possible association [153,154,159]. One possible explanation for the significant variations among the studies is the method used for virus detection. Polymerase chain reaction (PCR) is the most widely used technique among the studies, with the highest sensitivity for HPV DNA detection; however, in-situ hybridization (ISH) and immunohistochemistry (IHC) have also been used. Another reason for the high heterogeneity across studies is the ophthalmic tissues used to detect HPV DNA, as the prevalence of HPV DNA detection is higher in fresh tissues than in formalin-fixed paraffin-embedded (FFPE) tissues [160]. The higher rate of DNA degradation in FFPE tissues might be the reason for the variations. The prevention of sporadic RB due to HPV is similar to the prevention of HPV and other STDs. Barrier contraception and vaccination can avoid HPV and HPV-related malignancies, including RB [157].

### 4.5. Conjunctival Papilloma

Conjunctival papilloma is a slowly progressive benign tumor of the conjunctiva and is known to be responsible for 1–10% of the conjunctival lesions in children and adolescents [161]. However, its prevalence is higher in sexually active adults aged 20–40 [161]. The transmission route in infants is mainly through vaginal delivery; however, direct autoinoculation and sexually transmission are the predominant transmission routes in older children and adolescents [161,162]. Furthermore, low-risk HPV 6 and 11 were mostly detected in positive samples from conjunctival papilloma, while high-risk HPV 16 and 18 were the most common reported types in RB samples. The number of conjunctival papillomas in each eye is more likely higher in children and adolescents than adults [163]. Contrary to the controversial association between HPV and RB, HPV is a known risk factor for conjunctival papilloma development [161]. The oncogenic properties of HPV might be responsible for the changes that occur during conjunctival papilloma development [161]. Similar to the interaction of E7 and RB, E6 is the other encoded HPV protein and is associated with impairing the function of another tumor suppressor gene, p53 [162]. According to the “Two-hit” theory, both copies of the p53 gene should be impaired to allow for the progression of the tumor [162]. It is thought that the UV radiation is responsible for the first hit, and the HPV-associated oncogenesis is accountable for the second hit [162]. Histopathologic evaluation of the tumor revealed a fibrovascular core covered by papillary projections [161,163]. Sessile and pedunculated papilloma are more likely to be observed in adults and pediatrics, respectively [161]. The clinical implication of this observation is the lower probability of pedunculated papilloma to develop epithelial dysplasia [163]. It should be noted that patients with conjunctival papilloma are more likely to develop laryngeal papilloma [164,165]. Different treatments have been suggested for conjunctival papilloma, which are categorized into sole and combination therapies. Sole therapies are oral cimetidine (300–400 mg 3 times a day), photodynamic therapy, cryotherapy, and topical interferon alfa-2b (1 MU 3 times a day). The combination therapies are excisional biopsy and cryotherapy, excisional biopsy and cryotherapy and oral cimetidine, and excisional biopsy and cryotherapy and interferon alfa-2b [161].

## 5. Conclusions

HPV infection could lead to severe complications in mothers and their children. The worldwide prevalence of HPV among pregnant women is high, affecting mothers and their children in the short- and long-term. HPV positivity and certain adverse pregnancy outcomes are significantly associated with each other. A wide range of manifestations for children, from infancy to childhood, have been reported to be related to maternal HPV infections. However, as a curative treatment is not yet discovered for this infection, vaccination is the most feasible way of prevention.

## Figures and Tables

**Table 1:** Studies regarding maternal–fetal transmission of HPV.

Author, Year, Country	Features of the Participants of the Studies	Number of Pregnant Women Tested/Number of Positive Pregnant Women	Number of Newborns Tested/Number of Positive Newborns	Time and Sample for HPV Detection in Neonate	Type of HPV Screened (DNA PCR)	Positive Neonates/Positive Mother of Total Vaginal Delivery	Positive Neonates/Positive Mother of Total Cesarean Section
Trottier et al., 2016, Canada [27]	Pregnant women in the first trimester	167/75	67/7	At birth/3-month visits, conjunctival, oral, pharyngeal and genital areas	6, 11, 16, 18, 26, 31, 33, 34, 35, 39, 40, 42, 44, 45, 51, 52, 53, 54, 56, 58, 59, 61, 62, 66, 67, 68, 69, 70, 71, 72, 73, 81, 82, 83, 84, 89	-/- of 136	-/- of 31
Sánchez-Torices et al., 2015, Spain [40]	HPV-positive pregnant women	91/91	92/53	Immediately after delivery, cord blood and oropharynx	6, 11, 16, 18, 31, 33, 35, 38, 52	53/91 of 92	None
SkoczyNski et al., 2015, Poland [41]	Healthy pregnant women prior to delivery with a singleton pregnancy	152/29	152/16	Immediately after delivery, oral area	33 different HPV genotypes including 16 and 18	NM	NM
Hahn et al., 2015, South Korea [42]	Pregnant women over 36 weeks of gestation	469/72	469/15	Immediately after delivery, oral area and secretions	6, 11, 16, 18, 30, 31, 32, 33, 35, 39, 40, 42, 43, 44, 45, 51, 52, 53, 54, 55, 56, 58, 59, 62, 66, 67, 68, 68a, 69, 70, 72, 81, 82, 84, 90, 91	14/51 of 300	1/21 of 169
Lee et al., 2013, South Korea [14]	Healthy women with a singleton pregnancy	153/37	153/8	Immediately after delivery, cord blood and nasopharyngeal aspirate	6, 11, 16, 18, 26, 30–35, 39, 40, 42–45, 51–56, 58, 59, 61, 62, 66–70, 72, 73, 81–84, 90, 91	3/- of 108	5/- of 45
Hong et al., 2013, China [43]	HPV-positive pregnant women at delivery	3139/422	233/35	<24 h after birth, exfoliated oral and genital cells	6, 11, 16, 18, 33, 43, 56, 58	19/136 of 136	16/97 of 97
Park et al., 2012, South Korea [44]	Pregnant women over 36 weeks of gestation	291/55	291/10	Immediately after delivery, oral area	24 different HPV genotype including 6, 11, 16, 18, 31, 33, 35, 39, 40, 44, 45, 51, 53, 56, 58, 66, 68, 70	10/- of 193	None/- of 98
Koskimaa et al., 2012, Finland [35]	Pregnant women in third trimester of pregnancy	329/NM	331/59	At birth and till 2 months, oral area	6, 11, 16, 18, 26, 31, 33, 35, 39, 42, 43, 44, 45, 51, 52, 53, 56, 58, 59, 66, 68, 70, 73, 82	NM	NM
Smith et al., 2010, USA [45]	Healthy women with a singleton pregnancy	333/99	333/5	At a median of 42 h after birth, oral and genital areas, and cord blood	6, 11, 16, 18, 31, 33, 39, 51, 53, 54, 56, 58, 59, 61, 69, 66, 70, 83, 84	3/86 of 295	2/13 of 38
Castellsagué et al., 2009, Spain [38]	Pregnant women with potential risk of HPV exposure	143/66	143/26	From birth till 24 months of age, oral and genital areas	6, 11, 16, 18, 31, 33, 39	22/- of 124	4/- of 19
Rombaldi et al., 2008, Brazil [26]	HPV-positive pregnant women at delivery with prior history of HPV infection, abnormal smear, or genital warts	49/49	49/11	Immediately after delivery, oral, body, nasopharyngeal aspirates and cord blood	6, 11, 16, 18, 31, 33, 42, 52, 58	5/24 of 24	6/25 of 25
Gajewska et al., 2005, Poland [46]	Pregnant women with and without pregestational insulin-dependent diabetes mellitus	45/12	45/9	48 h after birth, oral area, cord blood	6, 11, 16	NM	NM
Rintala et al., 2005, Finland [47]	Pregnant women in the third trimester	76/57	77/56	At birth till 2 years, oral and genital areas	16, 18, 31, 33, 35, 39, 45, 51, 52, 54, 56, 58	-/- of 63	-/- of 13
Worda et al., 2005, Austria [48]	Pregnant women underwent cesarean section between 37 and 40 weeks of pregnancy	153/56	NM	NM	6, 11, 16, 18, 31, 33, 35, 39, 42, 43, 44, 45, 51, 52, 56, 58, 59, 68	None	-/56 of 153
Deng et al., 2005, China [49]	Pregnant women without condylomata acuminata in the genital tract	116/42	116/10	4 h after birth, cord blood, oropharyngeal secretions, amniotic fluid	6, 11, 16, 18, 31, 33	NM	NM
Bandyopadhyay et al., 2003, India [50]	Term pregnant women without a history of abnormal smears or genital warts	135/38	135/14	After birth, oral areas	6, 11, 16, 18, 31, 33	3/11 of 59	11/27 of 76
Peng et al., 2000, China [51]	Pregnant women in third trimester of pregnancy	31/23	31/13	Immediately after delivery, nasopharyngeal aspirates	6, 11, 16, 18	NM	NM
Tenti et al., 1999, Italy [52]	Pregnant women with negative Papanicolaou smear at first trimester	711/37	711/11	Immediately after delivery, nasopharyngeal aspirates	6, 11, 16, 18, 33	11/29 of 557	0/8 of 154
Wang et al., 1998, China [29]	Pregnant women on third trimester of pregnancy	73/26	73/11	Immediately after delivery, nasopharyngeal aspirates and amniotic fluid	16, 18, 35	7/14 of 49	4/12 of 24
Tseng et al., 1998, Taiwan [53]	Healthy women with a singleton pregnancy	301/68	68/27	At least 3 days after delivery, oral and genital area	16, 18	18/35 of 160	9/33 of 141
Xu et al., 1998, China [54]	Pregnant women on third trimester of pregnancy	30/16	30/14	12–48 h after birth, pharyngeal secretions	6, 11, 16, 18, 31, 33, 35, 38	- of 17	- of 13
Watts et al., 1998, USA [55]	Pregnant women before 20 weeks of gestation	151/112	151/8	At birth and till 3 years, oral, genital and anal areas, nasopharyngeal aspirates	6, 11, 16, 18, 31, 33, 35, 39, 45	6/- of -	2/- of -
Puranen et al., 1997, Finland [56]	Pregnant women	105/41	106/39	Immediately after delivery, nasopharyngeal aspirates	2, 6, 7, 11, 16, 18, 30, 31, 33, 53, 66	30/30 of 78	9/11 of 27
Alberico et al., 1995, Italy [57]	Pregnant women in the first trimester	170/53	170/37	Immediately after delivery, oropharyngeal secretions	6, 11, 16, 18, 31, 33, 52	NM	NM
Cason et al., 1995, United Kingdom [58]	Pregnant women, some of them had history of abnormal smears and genital warts	61/45	62/33	After birth, oral and genital areas, nasopharyngeal aspirates	16, 18	NM	NM
Parkarin et al., 1994, United Kingdom [59]	Pregnant women, some with a history of abnormal smears or of previous genital warts	31/20	32/12	24 h after birth, oral and genital areas	16, 18, 31, 33	NM	NM

Abbreviations: DNA: deoxyribonucleic acid; NM: not mentioned; PCR: polymerase chain reaction.

## Data Availability

Not applicable.
